# Supplementary material for: Whole-brain neural substrates of behavioral variability in the larval zebrafish
Source: bioRxiv. 2024 Mar 6:2024.03.03.583208. Preprint. [Version 1] doi: 10.1101/2024.03.03.583208 (PMC10942351; doi:10.1101/2024.03.03.583208)
Supplement: 1 [file NIHPP2024.03.03.583208v1-supplement-1.pdf]

bioRxiv preprint doi: https://doi.org/10.1101/2024.03.03.583208; this version posted March 6, 2024. The copyright holder for this preprint (which was not certified by peer review) is the author/funder, who has granted bioRxiv a license to display the preprint in perpetuity. It is made available under aCC-BY 4.0 International license.

Supplementary Figure S3, related to Figure 3, trial-to-trial variability in visually-evoked neurons is largely orthogonal to visual decoding dimensions

A. Schematic of trial-to-trial noise mode identification. First, dimensionality reduction is applied to the whole-brain dynamics (left) to identify the neuronal activity patterns that optimally preserve visual information using partial least squares regression. Within these visually-evoked neurons, each trial's average activity during the visual presentation is computed (middle, each dot represents a single trial corresponding to the pink or green stimulus condition). Finally, the covariance across trials is decomposed to find the noise modes $\vec{e}_{\alpha}$, which represent orthogonal directions within the visually-evoked neurons and ranked in order of decreasing variance.

B. $\vec{w}_{opt}$ robustly encodes the visual stimulus. Shown are example time traces of the stimulus kernel (blue, where the size of the stimulus is encoded by the magnitude and which visual field it is presented in is encoded by the sign) and the neural projection onto $\vec{w}_{opt}$ (orange), which exhibit a correlation of $r=0.88$, $p<10^{-6}$.

C. The largest brain-wide principal component (PC) encodes motor behavior. (i) Example time traces of the tail vigor (blue) and PC 1 (orange), which exhibit a correlation of $r=0.51$, $p<10^{-6}$. (ii) The coefficients of the neurons contributing most to the PC, which are distributed across the brain and are nearly all positively correlated with tail vigor.

bioRxiv preprint doi: https://doi.org/10.1101/2024.03.03.583208; this version posted March 6, 2024. The copyright holder for this preprint (which was not certified by peer review) is the author/funder, who has granted bioRxiv a license to display the preprint in perpetuity. It is made available under aCC-BY 4.0 International license.

Supplementary Figure S4: related to Figure 4, pre-motor neuronal populations predictive of single-trial behavior.

A. Classification of turn direction as a function of time window analyzed. The mean F score across n=7 larvae is used to assess the performance of multi-class classification of the visual stimulus. Top: a time window is swept across various start timepoints (y-axis) and end timepoints (x-axis) to determine the classification performance across various time intervals of the neuronal dynamics. Bottom: Mean $\pm$ 95% confidence interval of the F score for the best time interval ending at the given timepoint (Data), compared to shuffled data in which the stimulus labels are randomized. The classification accuracy is significantly higher than shuffled data ($p<0.05$, paired t-test) only after stimulus onset.

B. Turn direction classification as a function of stimulus size. On average before stimulus onset (blue line), the F score is highest for the smallest size stimuli, whereas the F score is relatively constant at the time of movement initiation (orange line, mean $\pm$ 95% CI across n=5 larvae).

C. As in A, except for classifying left or right turn direction from the projections onto the trial-to-trial noise modes $\vec{e}_\alpha$, as opposed to the whole-brain dynamics as used in Figure 4B.

D. As in A, except for classifying left or right turn direction from the projection onto the optimal stimulus decoding dimension $\vec{w}_{opt}$.

Video 1: related to Figure 2. Example target-directed and target-avoidance bouts in free behavior.

On the left, an example target-directed bout showing a 7dpf larval zebrafish approaching a stimulus presented on a screen below the dish (see Figure 2A). On the right, a similar target-avoidance bout from the same larva. The videos are slowed down 10x. These videos correspond to the composite images shown in Figure 2B-C.
